# The perspective of hypertension and salt intake in Chinese population

**DOI:** 10.3389/fpubh.2023.1125608

**Published:** 2023-02-17

**Authors:** Kexin Jiang, Tingting He, Yongzhi Ji, Tao Zhu, Enshe Jiang

**Affiliations:** ^1^Institute of Nursing and Health, Henan University, Kaifeng, China; ^2^Department of Basic Nursing, Henan Technical Institute, Zhengzhou, China; ^3^Department of Geriatrics, Kaifeng Traditional Chinese Medicine Hospital, Kaifeng, China; ^4^Department of Scientific Research, Scope Research Institute of Electrophysiology, Kaifeng, China

**Keywords:** hypertension, salt intake, dietary, salt reduction, Chinese population

## Abstract

Salt intake is too high nowadays. It has been widely recognized that there is a close relationship between hypertension (HTN) and dietary salt intake. Investigations reveal that long-term high salt intake, mainly sodium intake, induces a relevant increase in blood pressure in hypertensive and normotensive individuals. According to most scientific evidence, a diet with high salt intake in public increases cardiovascular risk, salted-related HTN, and other HTN-associated outcomes. Given the clinical importance, this review aims to present the prevalence of HTN and trends in salt intake in the Chinese population and will comprehensively discuss the risk factors, causes, and mechanisms of the association between salt intake and HTN. The review also highlights the education of Chinese people regarding salt intake and the cost-effectiveness of salt reduction from a global perspective. Finally, the review will emphasize the need to customize the unique Chinese practices to reduce salt intake and how awareness changes people's eating lifestyle and helps adopt diet salt reduction strategies.

## 1. Introduction

The human body needs a tiny amount of salt from food to maintain normal physiological functioning and fluid balance. In early times, the average salt intake for human ancestors was below 0.5 g/d, and the only source of salt was naturally found in foods at that time. However, the average salt intake is 10 g/d in most countries nowadays (1, 2), indicating a more than 20 times increase in a short period in the evolutionary timescale. Although the inventions of refrigeration technologies have obviated the need for salt as a preservative for food, salt is still considered the most taxed and traded commodity in the world (3). The increase in diet-salt intake is causing the public to become highly concerned because of its various adverse effects on human health and the inability of evolved human physiology to eliminate enormous amounts of salt. According to a recent report, a high salt diet was one of the top 3 dietary risk factors, which led to about 3 million deaths in 2017 (4). In daily life, table salt is mainly sodium chloride. It is one of the most active ions to maintain the continuity of normal physiological functioning of the human body and stabilize the fluid balance.

Hypertension (HTN) was defined as having systolic blood pressure ≥140 mmHg and diastolic blood pressure ≥90 mmHg by WHO, and this diagnosis criterion was used in China. However, according to current published literature, it has been firmly evaluated that sodium intake is the critical factor in determining blood pressure (BP) values (5–8). Furthermore, this high intake of sodium (>2 g sodium per day) and increase in BP values are associated with the onset of HTN and other related clinical outcomes like kidney damage and cardiovascular complications (9, 10). Therefore, considering sodium intake as the critical regulator for BP, restriction of sodium intake is recommended as a mandatory non-pharmacology measure to treat HTN (11, 12). Furthermore, a large volume of evidence has also suggested that restriction of sodium intake decreases BP levels and is associated with lessening HTN-linked complications and morbidity and mortality in cardiovascular disease (13).

Sodium intake is a dietary lifestyle risk factor for high-altitude cardiovascular disease in the Chinese population. This review examines the literature and cost-benefit analysis on high salt intake and the pathogenesis of HTN. The review has covered the literature on associated implications, mechanisms, impact, and subject awareness. Clinically, HTN is characterized by an elevated BP level, impacting health, disease, and death worldwide. HTN is the lifestyle associated disease (14–16). It is a non-communicable disease that is first treated in primary care and, if not treated properly at the time, can lead to various serious complications related to cardiovascular illnesses (45% mortality), renal failure, and death (17, 18). Findings demonstrate that even reducing just 5 mg of salt in daily diet routine will reduce 14% of stroke, 9% of heart disease, and 7% of all-cause mortality at a population level (19).

Due to economic development and population aging, HTN has become more prevalent globally, including in developing countries (20). For example, according to the latest National Health and Nutrition Examination Survey report, the number of HTN patients in US adults is increasing compared to previously reported data (21). Similarly, in Asian countries like Pakistan, India, and China, the prevalence of HTN is increasing with high confidence intervals and significant differences in prevalence between rural and urban populations (22, 23).

According to the six rounds of national survey reports, there were 274 million adults aged 18-69 years with HTN, and the standardized prevalence of HTN was 24.7% in 2018 in China (24). However, the awareness, treatment, and control levels of HTN were low at 38.3, 34.6, and 12.0%, respectively, in 2018. This situation demands improvement in detecting and treating HTN by strengthening primary care. Another cross-sectional study from a working population at high altitude in 2013 in China showed that the overall standardized prevalence and HTN and prehypertension were 26.7 and 41.3%, respectively. 36.5% were aware of HTN, 19.4% were being treated, and only 6.2% had their BP controlled (25). It indicates that this high prevalence of HTN and low awareness, treatment and control rates need to measure to improve this situation for these working populations at high altitude in China.

**Figure 1:**
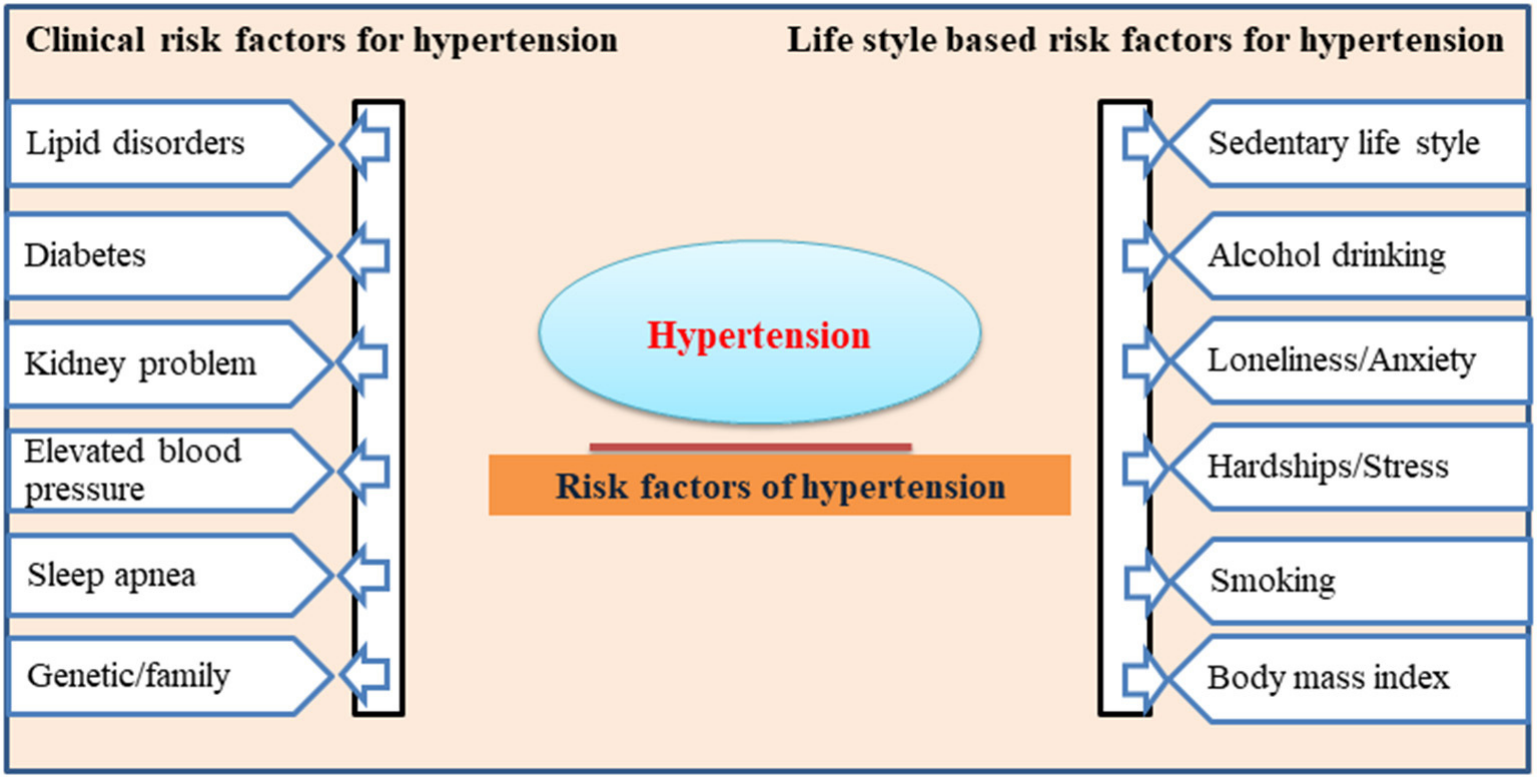
The clinical and lifestyle-based risk factors for hypertension development.

Many epidemiological studies show that age, gender, body mass index, waist circumference, sedentary lifestyle, smoking and alcohol intake are leading risk factors for the onset of HTN (26, 27). Other factors, including lipid disorders, diabetes, and a genetic family history of high BP, are the clinical complications leading to HTN (28). The clinical and lifestyle-based risk factors for developing HTN are listed in Figure 1. More recently, through a series of studies, it has been well established that excess salt intake plays a critical role in the pathogenesis of higher BP and the onset of HTN in the population (29). This clinical association between high salt intake and associated HTN development has drawn the attention of researchers globally to highlight the need to precisely evaluate these links and address the public to consider the necessary changes in their dietary lifestyle regarding salt intake in food.

## 2. Salt causes HTN

The Paleolithic humans consumed more salt (0.69 g of sodium per day) than our ancestors on an evolutionary scale (6, 30–32). Physiologists theorized that the discordance between present-day salt intake and what the human species is genetically programmed to handle had caused primary HTN. Given essential HTN, preliminary research on the association of salt intake and HTN found that the Alaskan Inuit population with a mean of 1.6 g sodium intake per day was free of HTN. However, 8.6% of Americans consumed a mean of 3.9 g sodium daily and were affected by HTN-related issues (33, 34). Other studies further explained that BP was higher in populations with higher sodium intakes, albeit with significant inter-group variability. For example, Urban Canadians had a 3.3 g salt intake daily. They had a median BP of 119/73 mmHg, compared to South Koreans who consumed over 4.6 g sodium daily and only had an associated BP of 109/71 mmHg (5, 35).

According to the data from the Prospective Urban Rural Epidemiology surveys, an epidemiological investigation involving over 100,000 individuals from 18 countries also supported there was a significant correlation between daily salt intake and BP (31, 36). Although there are limitations of differences in genetics between groups of these studies and a lack of a causative link between salt intake and BP, findings are suggestive. They concluded that there was a 2.11/0.78 mmHg increase in BP for every 1 g sodium intake increment. Studies from Poulter and Simmons further strengthen the rationale and provide supporting evidence that there is an increase in BP and sodium intake in urban migrants compared to the rural controls of the same ethnicity (37, 38). A recent meta-analysis on interventional trials for the effect of a low or high salt diet on BP found that the BP reduction was 3.47 mmHg for systolic BP and 1.81 mmHg for diastolic BP on <2 g/d vs. ≥ 2 g/d sodium intake (39). Another study supported by one of the most robust double-blind randomized crossover trials in 20 humans with untreated mild essential HTN reported a mean BP of 147/91 mmHg on 1.2 g/d sodium compared to 163/100 mmHg on 4.8 g/d (40).

### 2.1. BP elevation in response to salt-intake

The blood volume, heart rate and peripheral resistance of blood vessels determine the blood pressure level in the body. Therefore, HTN is closely related to the changes in these factors. A study showed high-salt diet increased the sodium concentration in plasma and cerebrospinal fluid in Dahl salt-sensitive hypertensive rats (41). The alteration of the plasma sodium concentration can cause water and sodium retention, resulting in hypervolemia, an increase in peripheral resistance of blood vessels and the evaluation of blood pressure. In addition, Neural mechanisms and endocrine secretions play a pivotal role in regulating BP. A high-salt diet can also induce the activation of neurons in the cardiovascular-related nucleus of the brain and the increase in sympathetic nerve activity (SNA). The central nervous system regulates sodium appetite and thirst, which will affect animal blood volume. Studies have shown that the activation of neurons in the hypothalamus releases vasopressin and orexin (42, 43). The increase of SNA caused by the high-salt diet increases peripheral resistance and activates the renin-angiotensin-aldosterone system in the body. These changes will cause further vasoconstriction and water and sodium retention, eventually resulting in HTN.

Physiologically, increased BP in response to overconsumption of salt is because of increased plasma volume. Researchers state that sodium retention increases serum sodium, increasing thirst and plasma volume and ultimately resulting in increased cardiac index and BP (40). Initially, the rising in cardiac index upon salt loading returned to a normal level. At the same time, total peripheral resistance (TPR) of the blood vessels will rise and remain elevated in old hypertensive adults (44). This association of salt intake-BP and HTN was elaborated in the finding of a study exhibiting the inverse relation of plasma volume to BP in patients with chronic HTN (45). These findings were further strengthened by comparing the hemodynamic between normotensive and hypertensive individuals. Results show that cardiac index was similar in both groups while total peripherial resistence was significantly elevated (46). Therefore, these studies establish that BP rise in chronic salt loading results from peripheral vascular constriction with other hemodynamic parameters remaining constant.

Another perspective on the BP elevation in response to excessive salt intake explains that plasma sodium may directly affect the vasculature. This cellular level minor elevations in sodium concentration increase the stiffness of isolated human endothelial cells of blood vessels, further facilitating the increase in BP (47). In addition, this rising in plasma sodium level due to a long-term high salt diet may also indirectly lead to systemic vascular constriction by changing sympathetic nervous system outflow (41, 48). These indirect effects have been well evaluated in rats, which occur via dietary sodium involvement in a hormonal pathway (49). From a hormonal pathway perspective, excessive salt intake leads to cardiac glycoside and marinobufagenin secretion from the adrenal gland, which increases BP (50). As a selective blocker for type 1 alpha subunit of the Na/K ATPase, Marinobufagenin works as digoxin (51). The release of these hormones leads to peripheral vasoconstriction and increased cardiac stroke volume, which causes an increase in BP (52). This elevation is mediated by the direct effects of sodium on the vascular endothelium and indirectly *via* nervous and hormonal pathways.

### 2.2. Sodium sensitivity by age, sex, and race/ethnicity

Salt-sensitive HTN is an increased blood pressure caused by a relatively high salt intake. The sensitivity to high salt intake is linked to people's age, gender, and ethnicity. Reports indicate that elevated BP may be related to sodium sensitivity due to an age-related decrease in renal sodium excretion (53, 54). Another study explained that increased BP response after dietary sodium intake correlates with age, especially for normotensive adults > 40 years old (55). A study showed that every 10 years of age is associated with a 2.4 mmHg increase in systolic BP in hypertensive patients with sodium sensitivity (56). Similarly, according to the GenSalt study, there is a 7.4 mmHg increase in systolic BP from low sodium to high sodium interventions in individuals with age ≥45 years old (*P* < 0.0001 for trends) (57). They further found that BP response to dietary sodium intake intervention is more prominent in women than men (58, 59). Physiologically, it is illustrated that female hormones might be related to increased renal sodium reabsorption and accompanied by water retention (60, 61). In addition, the genetic patterning and molecular findings suggest that genes encoding sex hormones could influence the sodium sensitivity of BP (62). It has also been found that sodium sensitivity also varies among different ethnic groups. In the published literature, it has been reported that sodium sensitivity is more common among individuals of African-American descent (59). Furthermore, Wright et al. (63) results also endorsed the prevalence of sodium sensitivity in black and white non-hypertensive but increased in hypertensive African Americans. This higher sensitivity in African American hypertensive might be because black individuals have an intrinsic reduction in the ability of renal sodium excretion compared with white individuals (64, 65).

## 3. Dietary salt practice in China and HTN

It is well known that the “king of all flavors,” salt, has been essential in preparing and preserving Chinese food for thousands of years. After thoroughly summarizing the studies about salt intake in China at the national, regional, and local levels in the near past, it has been concluded that most Chinese people consume excessive salt with their mean salt intake doubling the recommended upper limit. Related to this high salt intake, HTN patients are very much in the Chinese population, resulting in a significant burden of associated diseases in China (66). Although HTN onset is multifactorial, various Chinese population-based advanced studies explain that dietary salt has a vital role in the sensory properties of food. Therefore, dietary salt is highly associated with the rising burden of HTN in the ethnic group of the Chinese population (67).

Studies found that the older population of China is used to high salt intake in their daily dietary routine. For example, in a recent report, it was found that people from all provinces of China consume more salt than the recommended maximum intake of salt (5 g/d) and sodium (2 g/d) (68, 69). Similarly, in another systematic study, it has been concluded that the Chinese adult population consumes excessive salt due to low awareness about the high salt intake (70). A large-scale observational study on the Chinese population demonstrated that the mean sodium intake from three sites was 3.99 g/d (10.1 g/d salt), varying from 4.73 g/d (12.0 g/d salt) in northern China (Beijing and Shanxi province) to 2.49 g/d (6.3 g/d salt) in southern China (Guangxi province) (71, 72).

By using the 24-h urinary sodium measurement, researchers found that men were found to have more salt intake (12 g/d) than women (10.3 g/d) in 11 locations, including Guiyang, Guangzhou, Shanghai, Beijing, Shijiazhuang, and Taipei across China during the CARDIAC study (73). Furthermore, through exploring the relation of salt intake concerning regional and ethnicity references, it was found that Tibetans (14.8 g/d) and Kazaks (12.5 g/d) have more salt intake than Han (11.3 g/d) and Uygur (10.2 g/d) (74). Results of studies conducted in Beijing, Shanghai, and Guangxi also strengthen the previous findings that northern Chinese tend to have more salt intake (>14 g/d) than southern Chinese (< 9 g/d). It also demonstrates that men consistently have higher salt intake (8.8–17.2 g/d) than women (7.5–14.6 g/d) (75). Intraregional studies using the 24-h urinary sodium measurement indicate that salt intake is 11 g/d in Jiangsu Province, 13.9 g/d in Shandong Province, and 11.8 g/d in the city of Yantai (76, 77). Regarding salt sources, an INTERMAP study based on 4 day dietary recalls showed that about 75.8% of sodium intake for the Chinese population is from salt, which is added during home cooking (78).

Moreover, the latest results indicate that over 80% of Chinese people's sodium intake comes from salt added during the cooking process. On the contrary, most of the population's sodium intake in developed countries originates from processed food (79). Investigations also reveal that owing to decreased food taste with low salt, people of old age who may suffer from dysgeusia often face difficulty maintaining and following the lesser use of dietary salt and changing dietary practice and a salt-restricted diet (80). In addition, cultural food preferences may affect people's willingness to change a dietary habit (81). Furthermore, dietary practices may often represent an adult's cultural background and ethnic identity which further creates a challenge for serving staff in care units during the treatments (82). The published literature affirmed that salt intake in China exceeds the recommended thresholds and the body's physiological needs. These findings highlight the need to exert functional long-term national-level programs to reduce salt intake and raise the public's awareness level to adopt the trends on salt reduction in their dietary lifestyles.

### 3.1. Salt intake-related awareness in Chinese people

There are many salt reduction strategies with public education focusing on improving awareness of the people at risk associated with diets containing much sodium in food. The Chinese population is advocated to follow a healthy diet (83). The findings of different studies highlight the low level of formal education about high salt intake issues in Chinese people, which further affects their perceptions and adherence to related outcomes by over salt intake (82, 84, 85). Researchers also demonstrated that people have difficulty obtaining adequate education and knowledge about the adverse health effects of high salt intake. As a result, they have difficulties in acquiring effective salt reduction strategies. Other studies reported a need for knowledge among people of Northern China about the link between HTN and salt intake. 70% of the population is not aware of recommended upper limit of salt intake and associated damage, and 85.5% of members had never received formal salt-related health education (86, 87). Inappropriate education about health and salt intake lead to a misunderstanding about the effects of salt due to this health risk linked with salt intake remains to be revealed. Studies found that Chinese people wrongly believed salt was a source of energy and essential for the human body (88). Some people believed salt was particularly important if exercised, for it could replace the loss of sweat (89). These findings indicate the significant association between the general public's awareness of HTN and dietary salt reduction (cues to action). Furthermore, those aware of being hypertensive rarely translate this knowledge into action (88).

**Figure 2:**
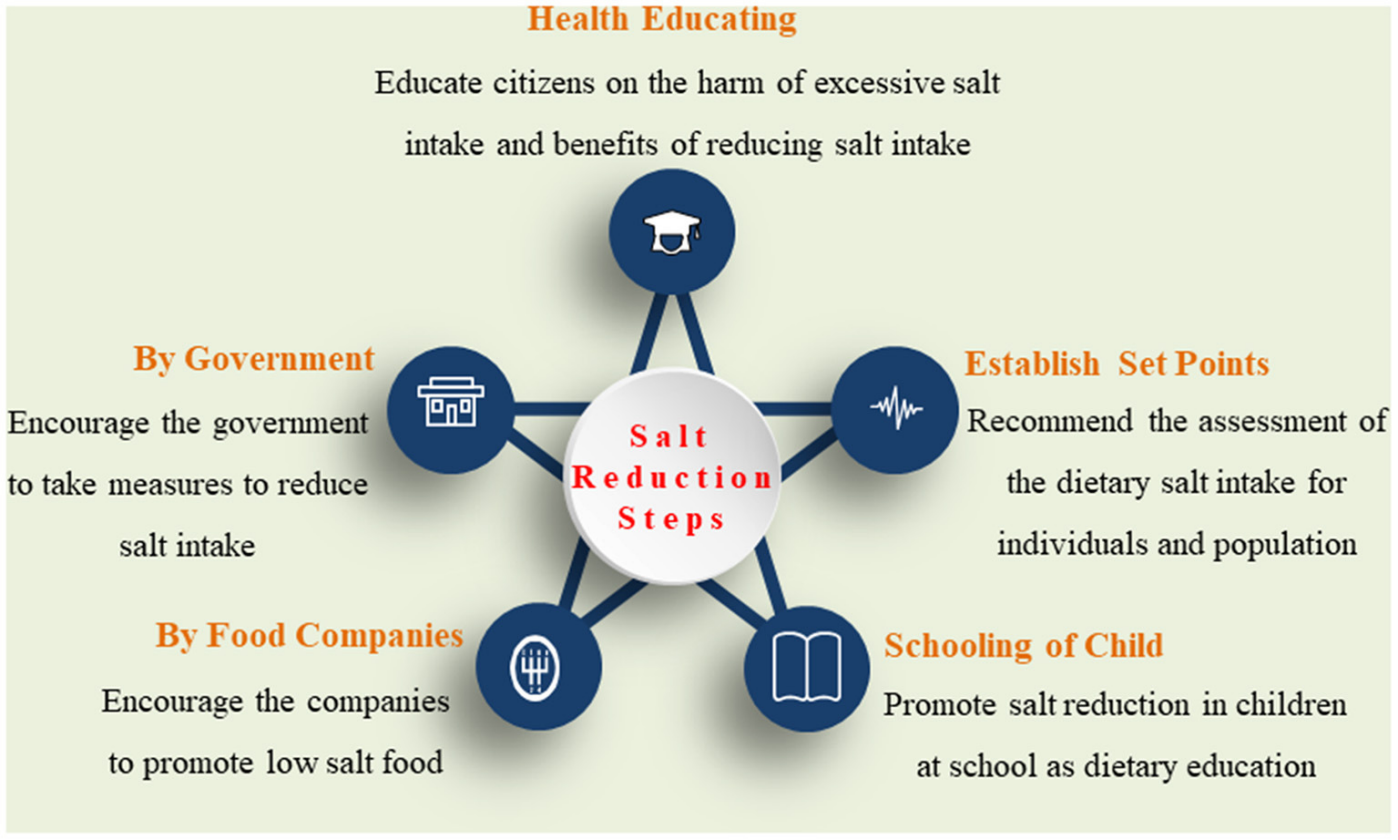
The recommended interventional strategies for salt reduction.

To better understand the excessive salt intake-related disease, it is crucial to recommend culturally appropriate strategies to address the population level and individual's perception of health risks and issues in salt-related behavioral changes in diet (Figure 2).

### 3.2. Cost-effectiveness of the population salt reduction

Cost-effective analyses from perspectives of international institutes have depicted that population-wide salt reduction companions have tremendously reduced premature deaths and CVD worldwide, including in high-income countries (HICs) and low- and middle-income countries (LMICs) (90–94). For example, the salt-reduction program implied by the United Kingdom has saved many lives from CVD, up to 9,000/year, and the annual financial budget for healthcare services is up to £1.5 billion (83). In the United States of America, 146,000 new CVD cases and deaths (more than 40,000) can be prevented by reducing salt intake to 3 g/d. Achieving this reduction in health can be comparable to lowering obesity or tobacco usage, saving 194,000–392,000 quality-adjusted life years and $10–24 billion in annual healthcare costs (95). The same goes for LMICs; a salt-reduction program must be more efficient or at least as effective as tobacco use to prevent CVDs (96, 97).

The main initiative has been taken to reduce people's salt intake. Studies found that in HICs, processed and fast foods account for 80 percent of salt consumption. The United Kingdom (UK) has reduced the salt content of numerous food goods by 20–50% over a decade. In the UK, all industries are encouraged to strive toward the same goals (83, 98). These results in a simultaneous drop in population salt intake, BP, and CVD death rate (2, 99). While some countries, such as Canada, Australia, and the United States, have adopted the voluntary salt objectives adopted by the United Kingdom (100, 101). South Africa and several other countries adopted the required salt targets (102), a more successful strategy. However, despite exceptionally high salt intake levels and these nations carrying more than 80% of the burden of salt-related diseases globally, salt reduction lags in most LMICs (103, 104). Salt awareness education is necessary to make people use less salt while preparing food at home. Although behavioral change is challenging, new and favorable strategies are being developed (105, 106). According to a study conducted in northern China, children may be crucial in encouraging the entire family to reduce on salt intake (107). Using salt alternatives, especially with less sodium and more potassium, has been demonstrated to lower mortality from CVD and HTN (106).

### 3.3. Chinese government salt reduction taskforce

To deal with the high salt intake and associated health damage, the Chinese government plays an essential role in reducing salt intake in its population. Especially the Chinese Center for Disease Control and Prevention is the key player responsible for making the salt reduction plan and implementing this program under the leadership of the National Health Commission of the People's Republic of China (74, 106, 108). In addition, other departments like the Ministry of Education, the Ministry of Transportation, and the State Administration for Market Regulation also contribute to creating a supportive national policy for salt reduction (108–110). The government must establish a Chinese Salt Reduction Taskforce comprising reputable individuals from various government ministries, industries, and academia. The taskforce's function is to spearhead and supervise the nationwide salt reduction efforts of the China Salt Reduction Initiative. In terms of the measures of salt reduction, Healthy China Action Promotion Committee issued the document of Healthy China Action (2019–2030) on July 9, 2019. The average salt intake per person less than 5 g/d as the goal of action on eating right was advocated and written in the document. The enterprise and supermarket were encouraged to participate in producing and selling low-sodium salt on the food counter. In 2019, the Chinese Center for Disease Control and Prevention designated the third week of September 15 each year as the “9.15 Salt Reduction Week”. A lot of low-salt diet knowledge is intensively disseminated to the public during this week. These measures encourage society to pay attention to and practice salt reduction actions. In addition, there should be clear and achievable goals set and monitoring systems to evaluate the progress toward the goals annually.

## 4. Conclusion

In conclusion, it is stated that overconsumption leads to several clinical complications that start from raised BP that further causes HTN and is independently associated with cardiovascular disease and mortality. This clinical association of salt intake, BP, and HTN is based on several physiological molecular and endocrine factors that facilitate the effect and impact on human health. Furthermore, associated damage from high salt intake may vary with the relative difference in age, gender, ethnicity and baseline BP levels. Therefore, the findings of this integrative literature review indicate the over-salt consumption in the Chinese population and the need for adequate attention to design new policies at the national level to initiate salt-related health education and programs for salt reduction in the diet. Although a few works have been done in this field, more extensive and better-designed researches are necessary to understand the optimal approach to enable the stakeholders to co-design culturally appropriate salted-related HTN education programs. In addition, further qualitative research will fill the knowledge gap regarding salt-related education and the challenges in producing dietary modifications among Chinese people.

## Author contributions

Conceptualization: KJ and EJ. Writing–original draft preparation: KJ and TH. Writing–review and editing: YJ and TZ. Funding acquisitions: TH, TZ, and EJ. All authors have read and agreed to the published version of the manuscript.
